# Biotransformation of Aliphatic and Aromatic Hydrocarbons by Aerobic Bacterial Strains Isolated from Brown Coal

**DOI:** 10.3390/ijms27031407

**Published:** 2026-01-30

**Authors:** Paulina Supel, Katarzyna Starzec, Piotr Kapusta, Joanna Brzeszcz, Paweł Kaszycki

**Affiliations:** 1 Department of Plant Biology and Biotechnology, Faculty of Biotechnology and Horticulture, University of Agriculture in Kraków, Al. Mickiewicza 21, 31-120 Krakow, Poland; paulina.supel@urk.edu.pl (P.S.); katarzyna.starzec.sd@student.urk.edu.pl (K.S.); 2 Department of Microbiology, Oil and Gas Institute—National Research Institute, Lubicz 25A St., 31-503 Krakow, Poland; piotr.kapusta@inig.pl (P.K.); joanna.brzeszcz@inig.pl (J.B.)

**Keywords:** lignite, autochthonous microorganisms, *Rhodococcus*, hydrocarbon xenobiotics, environmental biotechnology, bioremediation, biodegradation

## Abstract

Lignite collected from a brown coal deposit was colonized with fully aerobic bacteria exhibiting hydrocarbon biodegradation pathways. Six autochthonous strains were isolated and tested for tolerance and biotransformation potential towards various xenobiotics such as hexadecane, squalane, pristane, benzoic acid, naphthalene, phenanthrene, and diesel oil. After preliminary screening, four xenobiotic-resistant strains were selected (*Rhodococcus opacus* CUP11, *Pseudomonas fluorescens* CUP15, *Sphingobacterium* sp. CUP16, and *Rhodococcus* sp. CUP17) and further treated for 14 days under aerobic conditions with variant concentrations of each compound (1, 2.5, 5 and 10 g/dm^3^). Microbial population dynamics and xenobiotic level changes were monitored. *Rhodococcus opacus* CUP11 and *Rhodococcus* sp. CUP17 were the most metabolically versatile bacteria capable of biotransforming several xenobiotics. Among the best-performing strains, the highest degradation yields were obtained for CUP17 (81% removal of diesel oil applied at 10 g/dm^3^, 99% of 2.5 g/dm^3^ hexadecane and 27% of 1 g/dm^3^ squalane), and CUP11 (49% of 10 g/dm^3^ hexadecane and 48% of 1 g/dm^3^ pristane). The strain CUP16 utilized squalane (33% at 1 g/dm^3^). The results suggest that the lignite-indigenous bacteria may be applicable for bioremediation of persistent xenobiotics in environmental cleanup projects.

## 1. Introduction

Progressive urbanization, industrial development and increasing anthropogenic interference with the environment may contribute to the accumulation of xenobiotic substances in water, air or soil. Among them are hydrocarbon pollutants known to disturb the ecological balance due to their toxic and carcinogenic properties, which makes their elimination particularly necessary [1]. Conventional methods commonly used in environmental reclamation are based on physical and chemical approaches. However, these techniques are expensive and often do not lead to complete removal of pollutants but rather may result in transformation into new compounds, still potentially hazardous [2,3]. Therefore, biological remediation is currently receiving increasing attention as it offers a sustainable, environmentally friendly and efficient alternative for pollutant degradation [4,5]. Bioremediation involves processes of enzymatic degradation of various pollutants, or their conversion into less toxic forms with the use of microorganisms [1]. Bioremediation in situ can be carried out by stimulation of growth and metabolic activity of indigenous microbiota. In cases where autochthonous microorganisms are lacking or found incapable of contaminant biotransformations, bioaugmentation may serve as a feasible solution. The latter process consists of the introduction of properly selected allochthonous microorganisms to contaminated sites to facilitate pollutant degradation [1,4,6,7,8].

The rate of biodegradation and/or assimilation of a given xenobiotic depends not only on its chemical structure, concentration and toxicity, but also on physicochemical properties of the environment such as pH, temperature, humidity, availability of organic matter, and, most importantly, the diversity and abundance of microorganisms capable of pollutant transformation [9]. For that reason, effective bioaugmentation requires application of the selected microorganisms at relatively high biomass densities. However, excessive concentrations of toxic substances can completely inhibit microbial proliferation. In addition, not all chemical compounds can undergo bioremediation, especially the water-insoluble ones exhibiting limited bioavailability [10,11]. Thus, to enhance efficiency of the process, microorganism candidates should be carefully selected to ensure they reveal specific features enabling them to adapt to a toxic microenvironment.

Microbial resistance to specific, recalcitrant substances is often achieved via production of enzymes involved in xenobiotic degradation pathways [8,12,13,14]. Some microorganisms can also thrive in toxic environments due to their ability to regulate membrane fluidity by altering the lipid composition upon the presence of specific substrates such as polycyclic aromatic hydrocarbons (PAHs) [15,16]. Several bacterial and fungal strains, including *Bacillus* [17], *Pseudomonas* [17,18], *Rhodococcus* [17,19], *Acinetobacter* and *Candida* [17], are known to produce biosurfactants to reduce surface and interfacial tension [17,20], which facilitates their growth in hydrocarbon-rich environments.

In order to identify and select the most effective microorganisms suitable for bioaugmentation, a number of preliminary tests need to be conducted. High efficiency of contaminant decomposition should be further verified and confirmed by additional qualities that determine resilience to new environments. At the same time, the candidate strains must be entirely harmless to humans [21,22]. So far, the ability to biodegrade various hydrocarbon xenobiotics has been observed in bacterial strains belonging, among others, to the genera *Pseudomonas*, *Rhodoccocus*, *Streptomyces*, *Sphingomonas*, *Alcaligenes*, *Shewanella*, *Acinetobacter* or *Mycobacterium* [3,4,5,23,24,25,26].

Currently, researchers tend to focus on the search for microorganisms revealing unconventional trophic pathways that result in their exceptional adaptability to specific or extreme conditions. They can be isolated from various sites, especially from habitats rich in complex organic matter. Among such opportune cases are brown coal deposits that typically contain large amounts of organic substances derived from the decomposition of plant and animal matter. Brown coal occurs predominantly in the form of lignite which can be classified into three fractions: (i) water-soluble compounds, including humic acid, fulvic acid and polyphenols; (ii) organic solvent-soluble compounds, such as hydrophobic bitumens, resins and waxes; and (iii) insoluble matrix, called humine [27,28].

Lignite was found to be colonized with rich and biodiverse indigenous microbiota, consisting mainly of anaerobic bacteria and archaeons [29,30,31]. Most of the published studies dealing with autochthonous strains focused on the problem of coal methanogenesis [30,32,33] or lignite biodegradation [29] with microbial consortia under anoxic or strictly anaerobic conditions. No attempts to isolate aerobic microorganisms from brown coal samples have been documented to date. Considering both the lignite microbial colonization and composition of organic fraction, we hypothesized that aerobic bacteria occur among the brown coal-native microbiota and that these microorganisms are capable of biotransformation of various hydrocarbons.

The aim of this study was to isolate and identify aerobic microorganisms inhabiting lignite deposits and to characterize their bioremediation potential against several hydrocarbons represented by selected linear and branched aliphatic, as well as mono- and polyaromatic compounds. Since efficient metabolism of such xenobiotics is contingent on unique enzymatic pathways it was expected that identification of biochemically versatile and robust strains would bring about novel findings, particularly valuable in terms of possible environmental applications of bioaugmented hydrocarbon remediation.

## 2. Results and Discussion

### 2.1. Bacterial Strains Isolated from Brown Coal

Isolation of bacteria from lignite samples, followed by cultivation in the presence of crude oil in fully aerobic conditions, resulted in obtaining a collection of six strains, designated as isolates “CUP” and numbered 1, 11, 12, 15, 16, and 17. After macroscopic and microscopic characteristics, the strains were subjected to identification with 16S rDNA sequencing and, additionally, with API32GN for CUP15. For the latter case, the 16S rDNA method yielded an unsatisfactory % of identity as *Pseudomonas fluorescens* (98.80%) whereas API32GN gave very good results as *Ps. fluorescens* with 99.9% of identity (Table 1).

### 2.2. Preliminary Tests (Activity of Cellular Dehydrogenases in the Presence of Xenobiotics)

All the isolated strains were subjected to preliminary screening to analyze biodegradation capabilities towards selected xenobiotics, representing aliphatic and aromatic hydrocarbons, all applied at initial concentrations of 1 g/dm^3^. The results of the TTC-based respiratory tests obtained after one-week incubation with the xenobiotics are presented in Table 2. All the control variants, i.e., bacteria cultivated on glucose, proved positive. During the screening, the strains CUP1 and CUP12 (marked with an asterisk in Table 2) showed significant growth inhibition after streaking onto plates and therefore they were excluded from further tests. The strains CUP11, CUP15, CUP16 and CUP17 were subjected to further analyses involving bioremediation of particular hydrocarbon compounds together with monitoring growth of microbial cultures.

### 2.3. Bioremediation Tests

The strain *Rhodococcus opacus* CUP11 revealed the most versatile xenobiotic metabolism and therefore it was tested using all the examined xenobiotics. The remaining isolates, namely *Pseudomonas fluorescens* CUP15, *Sphingobacterium* sp. CUP16 and *Rhodococcus* sp. CUP17, were treated only with those hydrocarbons which yielded positive results in the preliminary respiratory experiment (Table 2). The results of bioremediation potential of the isolates are shown in Table 3 where changes in xenobiotic concentrations are listed upon 14-day incubation with bacterial cultures. Note that the data are presented only for the cases in which significant xenobiotic removal rates were observed along with incubation time. Despite positive cell dehydrogenase activities in the screening test, the strain CUP15 did not efficiently remove hexadecane, nor did CUP11 degrade squalane.

Biotransformation of benzoic acid was analyzed only for *Rhodococcus opacus* CUP11, that is the only strain exhibiting respiratory activity in the presence of this compound (Table 2). Unexpectedly, the treatment with benzoic acid applied at the lowest concentration of 1 g/dm^3^ led to a total culture growth inhibition as early as after 3 days of incubation. Due to the observed toxicity effect, changes in concentration of this xenobiotic were not determined. Better biotransformation results might be expected upon incubation with lower doses of benzoic acid. Note that other bacteria, mostly of the genus *Pseudomonas* [34,35,36] and *Microccocus* [37], proved able to degrade this compound admixed at initial concentrations ranging from 0.8 to 2.5 g/dm^3^.

**Figure 1:**
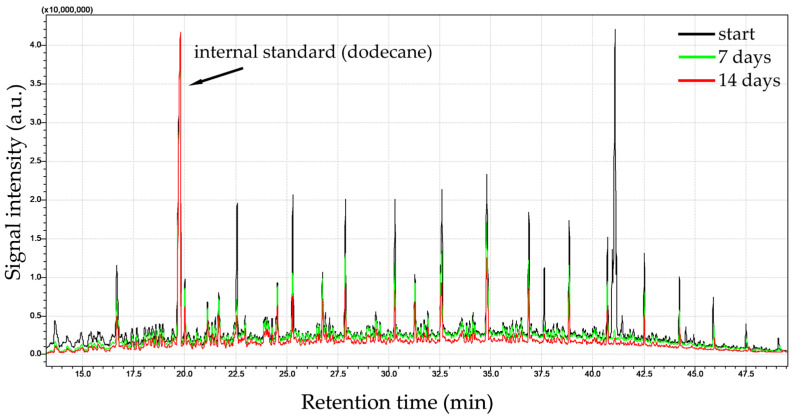
GC-MS analysis of diesel oil (initial concentration 10 g/dm^3^) biodegradation by *Rhodococcus opacus* CUP11.

**Figure 2:**
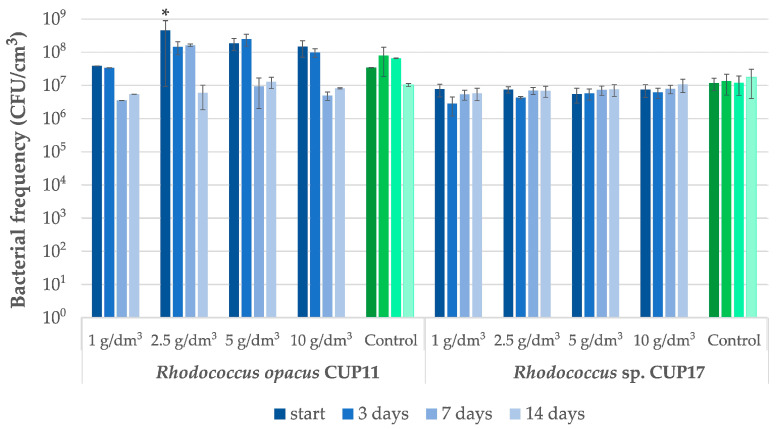
*Rhodococcus opacus* CUP11 and *Rhodococcus* sp. CUP17 cell frequency changes during the diesel oil bioremediation test. Control cultures were cultivated on glucose in the absence of diesel oil. An asterisk indicates statistically significant differences relative to control, assuming a significance threshold at *p* ≤ 0.01.

Diesel oil, a complex mixture of hydrocarbons, turned out to be a good source of easily available carbon for the strains *Rhodococcus opacus* CUP11 and *Rhodococcus* sp. CUP17 (Table 3). For both isolates, significant removal rates were obtained in most cases of the applied initial concentrations. In particular, the application of 1 g/dm^3^ of diesel oil led to approximately total biodegradation achieved within three days as determined relative to abiotic control (100% and 92.2%, for CUP11 and CUP17, respectively). Moreover, both CUP11 and CUP17 revealed considerable biodegradability determined after 14 days at higher initial diesel oil concentrations, i.e., 61.1% at 2.5 g/dm^3^ for CUP11 and 81.4% at 10 g/dm^3^ for CUP17 (see Table 3 for detailed data and Figure 1 for exemplary GC-MS analyses of CUP11 performance). At the same time no significant decrease in cell frequency was observed during incubation (Figure 2). Note that in the abiotic control samples the concentration of diesel oil also tended to decrease spontaneously, and almost 50% of the original pool was removed after 14 days. This effect was most likely caused by evaporation of low-density fractions. There are many reports documenting the in vitro biodegradation efficacy of bacteria towards diesel oil. Abdulrasheed et al. [38] showed the ability of *Arthrobacter* spp. isolated from Antarctica to degrade 36% of the initial diesel oil concentration of 5 g/dm^3^ after 7 days. In turn, *Pseudomonas aeruginosa* InaCC B290 and *Bacillus subtilis* InaCC B289 degraded diesel oil applied at 10 g/dm^3^ within 25 days of incubation with the yields of 36.5% and 37.5%, respectively [39]. *Cellulosimicrobium cellulans* and *Acinetobacter baumanni* were shown to remove 64.4% and 58.1% of the initial 20 g/dm^3^ concentration within 10 days [40]. Thi Pham et al. [41] documented 85% bioremediation of a mixture of 1.5 g/dm^3^ diesel oil, kerosene and gasoline with the *Rhodococcus* sp. Y2-2 strain. *Rhodococcus erythropolis* T7-2 isolated from mud contaminated with oil in Bohai Sea supplemented with nutrients such as yeast extract and mineral salts neutralized over 75% of diesel oil (10 g/dm^3^ initial concentration) under optimized conditions in 8 days [42]. However, the same strain cultivated in minimal medium was able to degrade only 15% of contaminants during the same period.

**Figure 3:**
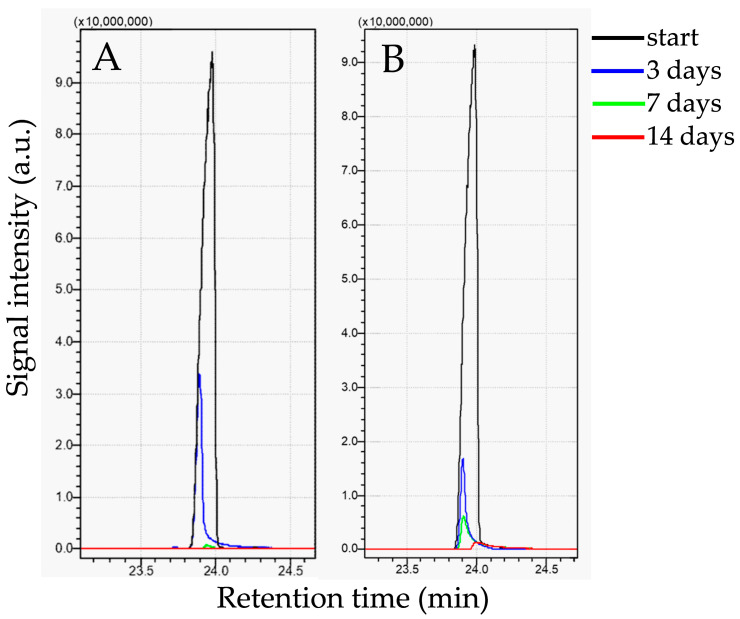
GC-MS analysis of hexadecane biodegradation by *Rhodococcus opacus* CUP11 (**A**), and *Rhodococcus* sp. CUP17 (**B**). Xenobiotic initial concentration: 1 g/dm^3^.

**Figure 4:**
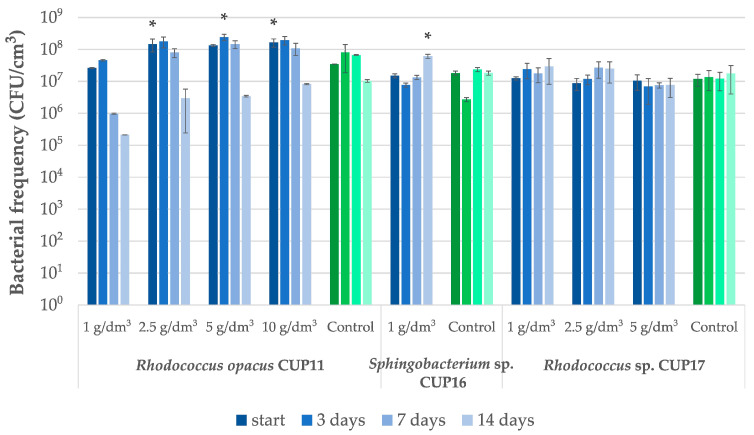
*Rhodococcus opacus* CUP11, *Sphingobacterium* sp. CUP16 and *Rhodococcus* sp. CUP17 cell frequency changes during hexadecane bioremediation test. The control cultures were cultivated on glucose in the absence of hexadecane. Asterisks indicate statistically significant differences relative to respective controls, assuming a significance threshold at *p* ≤ 0.01.

Satisfactory biodegradation results were obtained for hexadecane (Table 3). For *Rhodococcus opacus* CUP11 and *Rhodococcus* sp. CUP17, at initial hexadecane concentration of 1 g/dm^3^, almost complete removal was observed after 7 days (98.5% and 99.0%, respectively; Figure 3). Both strains revealed high remediation efficiency also at higher concentrations. For the best-performing strain CUP11, the removal rate reached 48.7% after 14 days at 10 g/dm^3^ initial concentration, whereas CUP17 proved to be able to remove 61.2% of hexadecane applied at 5 g/dm^3^. *Sphingobacterium* sp. CUP16 exhibited considerably lower degradation yield (22.9% at 1 g/dm^3^). However, no toxic effect of hexadecane was observed upon microbial frequency analyses of all the examined strains (Figure 4), which suggests that prolonged incubation of CUP16 might still reveal some biodegradation effect. A decrease in the cell count was observed for *R. opacus* CUP11 after the first week of treatment with 1 g/dm^3^ hexadecane and at the end of the experiment in variants with higher xenobiotic concentrations (Figure 4). This result can be explained by the depletion of the available carbon source due to its gradual assimilation (Table 3). Abdel-Megeed et al. [43] and Hristov et al. [44] analyzed hexadecane bioremediation by other strains belonging to the genus *Rhodococcus*. The first team demonstrated that at a starting concentration of 0.12 g/dm^3^, the resultant biodegradation rate of 85% was obtained within 6 days with *Rhodococcus erythropolis*. The authors also showed that hexadecane degradation by a three-strain consortium composed of *R. erythropolis, Pseudomonas putida,* and *Bacillus thermoleovorans* was more effective than the process carried out by each of these strains separately. In the study of Hristov et al. [44], *R. wratislawiensis* removed 100% of hexadecane supplemented at an initial concentration of 0.5 g/dm^3^ after only 12 h of incubation. Luong et al. [45] observed that actinobacterial strains *Rhodococcus erythropolis* X5 and S67 were capable of efficiently degrading high concentrations of n-hexadecane (2% *v*/*v*, that is 15.4 g/dm^3^). The strains removed hexadecane both in its liquid state at 26 °C (up to 53% degradation after 8 days) and the solid one at 10 °C (up to 40% degradation after 18 days), demonstrating remarkable cold-adaptation and hydrocarbon-assimilating capabilities of the bacteria tested. It is well known that some strains of the genus *Rhodococcus* and *Pseudomonas* can produce biosurfactants in the presence of hexadecane [46,47,48]. These substances reduce the surface tension, thus increasing the availability of the xenobiotic and bioremediation efficacy.

**Figure 5:**
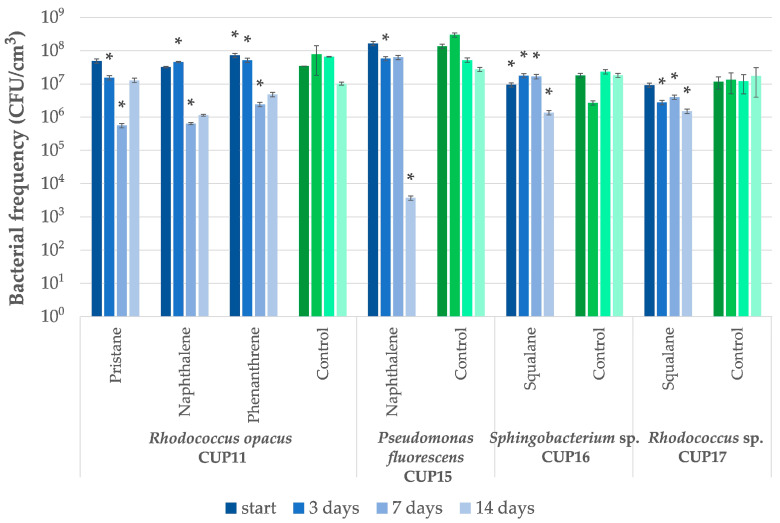
*Rhodococcus opacus* CUP11, *Pseudomonas fluorescens* CUP15, *Sphingobacterium* sp. CUP16 and *Rhodococcus* sp. CUP17 cell frequency changes during bioremediation test performed for selected hydrocarbons: pristane, naphthalene, phenanthrene and squalane, applied at initial concentrations of 1 g/dm^3^. The control cultures were cultivated on glucose in the absence of a given xenobiotic. Asterisks indicate statistically significant differences relative to respective controls, assuming a significance threshold at *p* ≤ 0.01.

Preliminary tests with a branched hydrocarbon squalane demonstrated that four strains were capable of surviving in the presence of 1 g/dm^3^ of this compound (Table 2). The subsequent experiment (Table 3) revealed a limited biodegradation potential of squalane admixed at 1 g/dm^3^, observed only for *Sphingobacterium* sp. CUP 16 and *Rhodococcus* sp. CUP17, whose removal yield reached 33.1% and 27.0% respectively. At the same time, both strains were able to proliferate in the presence of this hydrocarbon (Figure 5). Although *R. opacus* CUP11 was earlier identified among bacteria exhibiting biochemical activity in the presence of squalane (Table 2), no significant changes in hydrocarbon concentration were detected upon bioremediation test. Berekka and Steinbuchel [49] investigated three strains of different *Rhodococcus* species: *R. ruber, R. rhodochrous* and *R. opacus*, and reported only slight bacterial growth in media containing squalane as a sole carbon source. In the same study, the ability to proliferate in the presence of this hydrocarbon while utilizing it was demonstrated for two strains: *Mycolicibacterium fortuitum* NF4 and *Mycobacterium ratisbonense* SD4, which achieved 44% and 97% squalane removal, respectively, after one week of treatment at an initial concentration of 5 g/dm^3^. Similarly, *Gordonia amicalis* HS-11 exhibited 80% biodegradation of squalane applied at the same concentration within eight days [50]. More than 50% utilization of this xenobiotic was observed for *Arthrobacter* sp. NJ5 strain after only two days of incubation; however, this experiment was conducted at a substantially lower initial concentration of 0.2 g/dm^3^ [51].

Pristane, another branched hydrocarbon, was reported to be particularly difficult to biodegrade. Therefore, it is often used as an internal standard in studies of persistent alkane degradation [52,53]. In our study, from among the tested strains, only *Rhodococcus opacus* CUP11 proved to be able to reveal dehydrogenase activities in the presence of 1 g/dm^3^ pristane (Table 2). Then, the strain was capable of degrading this compound initially applied at 1 g/dm^3^ at a yield of 47.8% after 14-day incubation, compared to changes in the control (Table 3). Although statistically significant decrease in microbial frequency was observed after 3 and 7 days of incubation, at the end of the test bacterial population density equaled that of the untreated control (Figure 5). For all cases with higher initial concentrations applied, biodegradation was not observed. To date, a few bacterial strains, including *Rhodococcus*, were shown to utilize pristane as a sole carbon source. Some studies reported significant degradation efficiencies, exceeding a 50% removal of the initial concentration of 5 g/dm^3^ after three weeks for *R. ruber* [54], 40% and 15% degradation rates after 7 days for the strains *Rhodococcus* sp. T13 and TMP2, respectively (initial concentration of 1.25 g/dm^3^) [55], and 64% removal rate obtained for *Rhodococcus* sp. p52 after 9 days (initial concentration 0.2 g/dm^3^) [56]. It is of note here that pristane biodegradation was not limited to *Rhodococcus* species, only. For example, the strain *Alcanivorax hongdengensis* A-11-3 demonstrated capacity to utilize over 50% of pristane within seven days when exposed to a concentration of 10 g/dm^3^ [57].

Naphthalene biodegradation tests were performed using *Rhodococcus opacus* CUP11 and *Pseudomonas fluorescens* CUP15. When treated with 1 g/dm^3^ concentration of the compound, the CUP11 culture showed a 100-fold decrease in bacterial population after 7 days, whereas CUP15 suffered from a significant drop in bacterial abundance, recorded as four orders of magnitude after two weeks (Figure 5). Although a moderate toxicity effect of naphthalene cannot be excluded, these results rather point to the xenobiotic poor bioavailability due to low solubility in the aqueous medium (31.6 mg/dm^3^ at 25 °C). Despite the observed bacterial frequency decrease, both strains were able to effectively degrade naphthalene admixed at the concentration of 1 g/dm^3^, revealing the potential to remove 54.0% and 44.9% of the xenobiotic, respectively, relative to the abiotic control (Table 3). Other studies showed that bacterial degradation of naphthalene was possible only at substantially lower concentrations, such as 0.08 g/dm^3^ [58] or 0.3 g/dm^3^ [59]. *Pseudomonas* sp. DRK 9.1, isolated from volcanic mud in Renokenongo Village, Indonesia, demonstrated almost complete degradation of 20 mg/dm^3^ of naphthalene after four days [60]. In turn, *Paraburkholderia aromaticivorans* was reported to degrade 30 mg/dm^3^ of naphthalene within three days [61]. *Pseudomonas aeruginosa* was shown to remove this compound under anaerobic conditions, with biodegradation efficiencies reaching 65.5% and 33% at concentrations of 0.5 mg/dm^3^ and 20 mg/dm^3^, respectively [62]. Liu et al. [63] demonstrated that immobilizing *Microbacterium paraoxydans* on composite gel beads (carrier material) substantially enhanced biodegradation of naphthalene in water from 84% observed for free bacterial culture to 99% for the carrier-immobilized system, for initial concentration 200 mg/dm^3^. In the context of the above findings, the ability of the strains CUP11 and CUP15 to biotransform approximately half of the naphthalene pool upon treatment at 1 g/dm^3^, as documented in the present study, appears as an extraordinary feature, since to the best of our knowledge there are no other reports available which describe successful bioremediation of naphthalene applied at such high concentrations by a single strain cultured in a liquid medium.

For phenanthrene bioremediation experiments, *Rhodococcus opacus* CUP11 was the only strain that was selected after proving positive in the preliminary test. However, based on the two-week observations of biotransformation of the xenobiotic applied at 1 g/dm^3^, only modest degradation activity could be detected when compared to the abiotic control (15.7%; see Table 3). Microbial frequency analysis revealed about tenfold decrease of the cell number after seven days of incubation (Figure 5). As with naphthalene, such a result can be explained by a starvation effect caused by limited bioavailability due to extremely poor water solubility of phenanthrene (1.2 mg/dm^3^ at 25 °C). The majority of studies by other researchers on phenanthrene biodegradation were conducted at relatively low concentrations, typically well below 1 g/dm^3^ [64,65,66,67]. For example, *Burkholderia fungorum* FM-2 achieved nearly complete degradation of phenanthrene applied at initial concentration 0.3 g/dm^3^ and over 80% at 0.6 g/dm^3^ within two days [68]. More than 80% biodegradation of concentrations not exceeding 0.05 g/dm^3^ were also documented for *Arthrobacter* sp. P1-1 [69], *Arthrobacter* sp. YC-RL1 [70] and *Pseudomonas* sp. Ph6-gfp [71]. There are several other representatives of the genus *Rhodococcus* that demonstrated bioremediation potential toward phenanthrene, although the experiments were conducted applying low concentrations relative to our tests. Namely, for the strain *R. opacus* 0.2 g/dm^3^, naphthalene treatment resulted in a biodegradation rate of 52% [72], *Rhodococcus* sp. CMGCZ—0.1 g/dm^3^, degradation yield of 15% [73], *R. gingshengii* FF—0.05 g/dm^3^ with a 90% removal [74], and *Rhodococcus* sp. P14, for which a 43% degradation was observed at 0.05 g/dm^3^ [75]. Taking into consideration the data cited above and the weak bioavailability of phenanthrene, the ability of CUP11 to produce dehydrogenase activities (Table 1) together with the revealed biotransformation potential upon treatment with 1 g/dm^3^ of the compound should be regarded as a promising result.

Low solubility and physical states of the examined hydrocarbons were considered in more detail while arranging the experimental setup to trace bioremediation capabilities of bacterial isolates. Incubations of bacteria with particular xenobiotics were carried out in liquid media, in vigorously shaken (200 rpm) flasks, which ensured even dispersion of liquid hydrocarbons (diesel oil, hexadecane, pristane, and squalane), and favored the contact between bacterial cells and undissolved particles of naphthalene and phenanthrene. At the same time the tendency to microbial biofilm formation on the solids was minimized and we assumed that most of the bacteria remained in the suspension to allow for population density monitoring.

It should also be emphasized here that the experiments of this study were carried out under fully aerobic conditions. All tested strains proliferated and were biochemically active proving preferentially aerobic metabolism. Both culture growth and incubations during biodegradation tests were performed in non-sealed flasks allowing for free atmospheric oxygen penetration while aeration was provided by intensive and continuous mixing of cultivation media on shakers. Importantly, aerobic bioconversion of xenobiotics is considered the most time- and energy efficient [76]. The access to oxygen as a final electron acceptor in xenobiotic oxidations enables the course of many oxygen-dependent enzymatic pathways, for example mono- and dioxygenases in aromatic hydrocarbon transformations [70]. Based on the literature data, the isolates examined in this work belong to bacterial genera revealing predominantly aerobic growth and metabolism of organic pollutants [70,77,78,79,80,81,82]. Nevertheless, the genera *Arthrobacter* [83] or *Pseudomonas* [84] were shown to be capable of performing anaerobic nitrate respiration; however, this problem was not considered in this work.

## 3. Materials and Methods

### 3.1. Microbiological Material—Isolation and Identification of Bacterial Strains

Microorganisms were obtained from the lignite samples, collected from brown coal deposits located in south-western Poland. The lignite source material was described in detail by Szubert et al. [85] who studied anaerobic biogasification process for in situ methane production. The samples consisted of macerals of the dominating huminite group (84.4%), which included humodetrinite (50.6%), humotellinite (31.3%), and humocollinite (2.5%). The liptinite group (14.4%) was composed predominantly of sporinite and resinite. The content of total organic carbon (TOC) was 36.8% (*w*/*w*) and that of humic acids 1.6%. Within the extractable organic matter, the bitumen fraction contained 2.6% (*m*/*m*) of saturated (aliphatic alkanes n-C17 to n-C26, cyclic saturated terpenoids norphopanes, C20 phyllocladane, and C29 sterane) and 4.7% of aromatic hydrocarbons (including phenanthrene and its derivatives, di- and trimethylanphtalenes), as well as 34.9% resins and 57.8% of asphaltenes. The other lignite characteristics such as the content of dissolved organic carbon, specific surface area, petrographic data, and elementary analysis results are given in [85]. Aerobic bacterial strains were isolated from the internal, anaerobic layer of coal. The ground material was shaken for 1 h at 200 rpm in a buffer containing physiological saline (0.85% NaCl, Sigma Aldrich, Burlington, MA, USA) supplemented with sodium pyrophosphate (0.1%, *w*/*v*, Sigma Aldrich, Burlington, MA, USA) applied at a 1:9 (*v*/*v*) ratio. The resultant extracts were diluted with a modified Koch serial dilution method [86] and spread on the surface of a modified mineral Bushnell Haas (BH) (Sigma Aldrich, Burlington, MA, USA) with 15.0 g/dm^3^ Bacto™ Agar (BD DIFCO^TM^, Franklin Lakes, NJ, USA), 1 g/dm^3^ NaCl, final pH 7.0 ± 0.2 supplemented with 1.0 cm^3^ SL-10 trace element solution (Sigma Aldrich, Burlington, MA, USA). Crude oil (0.5 cm^3^ per Petri dish) was added as a sole carbon and energy source. Crude oil specifications: Zechstein, Main Dolomite lithostratigraphy; density: 816.3 kg/m^3^; kinematic viscosity: 4.22 mm^2^/s. The chemical content (wt.%): sulfur: 0.14; saturated hydrocarbons: 79.8; aromatic hydrocarbons: 15.3; resins: 4.4; asphaltenes: 0.5. After incubation for 20–30 days at room temperature, colonies growing in the presence of crude oil were selected and streak-passaged onto the Nutrient Agar (BD DIFCO^TM^, Franklin Lakes, NJ, USA), supplemented with 0.2% (*w*/*v*) sodium acetate (Sigma Aldrich, Burlington, MA, USA), to obtain pure cultures. Colonies of each strain were then characterized macroscopically (colony shape, color, etc.) and microscopically (cell morphology, motility, presence of spores, Gram staining) and subsequently identified using the API32GN (bioMérieux, Marcy l’Etoile, France) or 16S rDNA sequencing analysis (CB DNA, Poznań, Poland) as in [87]. Sequencing of 16S rDNA (for bacteria) was carried out on DNA extracted using a modified Marmur protocol, which applies phenol:chloroform:isoamyl alcohol in a 25:24:1 (*v*/*v*/*v*) ratio (all reagents from Sigma Aldrich, Burlington, MA, USA). PCR amplification employed the primer pair 27f (5′-AGAGTTTGATCCTGGCTCAG-3′) and 1492r (5′-GGTTACCTTGTTACGACTT-3′) [88] and was performed in a PCR thermocycler (Mastercycler Gradient Eppendorf, Hamburg, Germany) under the following profile cycles: an initial at 95 °C for 5 min, followed by 35 cycles of 95 °C for 30 s, 55 °C for 30 s, 72 °C for 1 min, and a final one at 72 °C for 7 min [89]. Sequencing products were determined using the BigDye Terminator v3.0 Ready Reaction Cycle Sequencing Kit (Amersham Biosystems, Freiburg, Germany) and analyzed on an ABI Prism 3100 Genetic Analyzer (Thermo Fisher Scientific, Waltham, MA, USA). Identification of the obtained sequences was conducted with the MicroSeq 16S rDNA database and the BLAST+ 2.17.0 tool [90]. The 16S rDNA sequences of the strains were deposited in the NCBI database. The API identification analysis was carried out for the *Pseudomonas fluorescens* CUP15 isolate with the use of API32GN standardized automatic identification system for Gram-negative rods, according to the producer’s manual [91].

### 3.2. Preliminary Tests—Metabolic Diversity Evaluation of Bacterial Isolates

Microbiologically pure strains used for metabolic diversity tests were cultivated in a liquid Standard Nutrient Broth (SNB) medium containing 3.0 g/dm^3^ yeast extract, 15.0 g/dm^3^ casein peptone (both from Biomaxima, Wrocław, Poland) and NaCl (6.0 g/dm^3^) on rotary shakers for 24 h at room temperature. The cultures were then centrifuged (4656× *g*, 10 min) and re-suspended in a liquid mineral BH medium not supplemented with any carbon source to obtain a final optical density OD_600_ = 1. In order to preliminary evaluate metabolic activities of the strains, a range of xenobiotics representing major groups of aromatic and aliphatic hydrocarbons were tested at 1 g/dm^3^ initial concentration, that is benzoic acid, naphthalene, phenanthrene, pristane, squalane and hexadecane (all from Sigma Aldrich, Burlington, MA, USA). Also, diesel oil was used as an additional variant to establish the strains’ potential for bioremediation of hydrocarbon mixtures. Due to very low amounts of the compounds needed for the microtiter plate-tests, xenobiotic stock solutions in hexane were prepared and transferred at given volumes to 0.2 cm^3^ wells of multi-well plates (Table 4), and then left for solvent evaporation. This allowed us to obtain the wells coated with the tested xenobiotics. As a positive control, the respective incubation variants containing 1 g/dm^3^ glucose (Sigma Aldrich, Burlington, MA, USA) were examined, while the negative control contained no carbon source.

Next, aliquots of 0.2 cm^3^ of bacterial cultures (OD_600_ = 1) in BH medium were transferred directly into the wells coated with the respective xenobiotics and incubated for three weeks in aerobic conditions on rotary shakers. The respiratory activity of each strain was assessed using the 2,3,5-triphenyltetrazolium chloride (TTC) method [92] after one week. A volume of 0.05 cm^3^ of 1% TTC (Sigma Aldrich, Burlington, MA, USA) solution was added to each well, and after 1 h incubation at room temperature in darkness the formation of red tetrazolium formazan (TF) precipitate, a product of activities of cellular dehydrogenases, was observed. The red color was assumed as representing bacterial metabolic activity in the presence of a given xenobiotic. After evaporation of water, 0.2 cm^3^ of methanol was admixed to dissolve the TF precipitate. The absorbance of the red solution of TF in methanol was measured spectrophotometrically at a wavelength λ = 484 nm. The result was marked as positive if the red solution color was detected. For further analyses, only these combinations of strains and xenobiotics were selected that yielded a positive reaction in at least three repetitions out of four samples analyzed.

### 3.3. Xenobiotic Biotransformation Tests

The selected strain-xenobiotic combinations with positive results of the preliminary assay were subjected to two-week biotransformation experiment. It was carried out in two stages: first, applying only one concentration of 1 g/dm^3^, and second, with higher concentrations, but only for combinations involving strains that showed significant xenobiotic degradation in the first stage.

Twenty-four-hour cultures pregrown in SNB medium were centrifuged, washed twice to remove residual carbon sources and resuspended in BH liquid medium to an optical density of approximately OD_600_ = 0.6. Meanwhile, pristane, hexadecane and diesel oil as well as the hexane stock solutions of naphthalene, phenanthrene and squalane were added directly to the Erlenmeyer flasks in appropriate aliquots to achieve variant final concentrations of 1, 2.5, 5 or 10 g/dm^3^ for each compound, assuming a total incubation medium volume of 23 cm^3^ (Table 4). The hexane solutions were left for solvent evaporation. Bacterial suspensions were introduced into the flasks coated with the xenobiotics. To minimize potential errors resulting from uneven xenobiotic distribution during sampling, the experiment was designed as a series of parallel flasks with identical culture-xenobiotic combinations, and at each time point three flasks were analyzed in their entire volume. An additional set of abiotic controls was also prepared, consisting of the sterile medium with a given xenobiotic applied at a concentration of 1 g/dm^3^. The flasks were covered with aluminum caps and incubated on rotary shakers at 200 rpm to provide fully aerobic conditions and facilitate direct contact of bacteria with xenobiotics. In several cases (see Table 3), these control sets revealed time-dependent concentration changes of particular organic compounds, which were caused by evaporation of either low-density xenobiotics or aqueous medium. These changes were taken into account in further data analyses, using the following formula to calculate biodegradation efficiency, based on the values of concentrations determined in particular samples, S, and abiotic controls, C:

Xenobiotic removal rate in (%) = (1 − (S_final/S_start)/(C_final/C_start))·100%

Bacterial growth was monitored using plate count method and the changes in xenobiotic concentration were analyzed chromatographically. The strains incubated in the presence of glucose served as positive controls, whereas those without a carbon source served as negative ones. In additional abiotic controls, sterile BH medium containing a given xenobiotic was examined to monitor concentration changes in the absence of microbial activities. Microbial frequency was monitored using the serial decimal dilution method. Diluted samples were spread on Petri dishes containing enriched agar (Biomaxima, Wrocław, Poland) applied as the standard growth medium. The plates were incubated at room temperature for 2–4 days. The number of bacteria was calculated as colony forming units (CFUs) per cm^3^ of the culture [86].

Xenobiotic concentration changes upon incubation with bacterial strains were analyzed using gas chromatography coupled with mass spectrometry (GC-MS) in hexane extracts obtained from microbial cultures with the use of Shimadzu GC-17A Gas Chromatograph coupled with a QP-5000 Mass Spectrometer and a Shimadzu AOC-20i Autosampler (Shimadzu Corp., Kyoto, Japan). A 60 m Equity-1 capillary column (Supelco, Bellefonte, PA, USA) packed with a non-polar dimethylsiloxane stationary phase and helium 5.0 as the carrier gas were used [87,93]. Prior to extraction, dodecane (Sigma Aldrich, Burlington, MA, USA) was added to the flasks as an internal standard to assess extraction efficiency. Due to the strong hydrophobicity and low solubility of xenobiotics, the extracts were prepared from the entire culture volume. For diesel oil the following column temperature program was applied: 50 °C (hold for 2 min), and then increase by 5 °C/min up to 330 °C (hold for 2 min). The injector and linker temperatures were 335 °C and 330 °C, respectively. The total separation time was 55 min, which enabled to detect the C8–C40 hydrocarbon range. For all other analyzed components, the temperature program was as follows: 50 °C (hold for 10 min), and 15 °C/min up to 320 °C (hold for 10 min). The injector and linker temperatures were 325 °C and 320 °C, respectively. The total separation time was optimized individually for each xenobiotic, and ranged from 25 to 28 min. One µL samples were injected automatically with an SGE precision microsyringe (SGE Analytical Science, Ringwood, VIC, Australia). The autosampler operated in splitless mode with sampling time set to 1 min [94]. Analyte quantification limits were established as 0.05 g/dm^3^, whereas the limits of detection (LOD) were determined separately for each xenobiotic and ranged from 0.001 to 0.005 g/dm^3^. Chromatograms were processed using GCMS-Solution 1.2 (Shimadzu Corp., Kyoto, Japan) and AMIDS GC.MS Analysis integrated with the NIST Mass Spectral Database 2014 (NIST, Gaithersburg, MA, USA).

### 3.4. Chemicals, Microbiological Media and Other Reagents

All xenobiotics and chemical substances used in the experiments, including salts, glucose, 10-SL solution elements, hexane, etc., were of analytical grade, and were purchased from Sigma Aldrich (Burlington, MA, USA). Microbiological media, if not specified otherwise, were obtained from Biomaxima (Poznań, Poland). Deionized water was used for preparation of aqueous solutions. Whenever required, fully sterile conditions were provided. Microbiological media, buffers and water were sterilized for 30 min at 121 °C in an autoclave.

### 3.5. Statistical Analysis

Preliminary bacterial screening test (Section 3.2) was carried out in quadruplicate. Bioremediation experiments (Section 3.3) involving particular combinations of a strain and xenobiotic were performed in three biological repetitions, that is three independent incubations in separate flasks. Microbiological frequency assessments in the culture samples were conducted in two technical replicates per flask. GC-MS analyses of hexane extracts obtained from each flask were done in two technical chromatographic runs. Whenever applicable, the results were analyzed statistically with the Statistica 13.3 (TIBCO Software Inc., Pao Alto, CA, USA) software. Significance of differences was evaluated with the one-way variance analysis (ANOVA) followed by Tukey’s post hoc test at *p* ≤ 0.05. Statistically significant differences between individual data are denoted by asterisks. The tabularized data regarding xenobiotic determinations with GC-MS were given as mean values ± standard deviations.

## 4. Conclusions

Lignite colonization with anaerobic microorganisms, as earlier documented, is due to the limited accessibility of atmospheric oxygen and predominately hydrophobic microenvironment. In this study, however, lignite samples collected at a brown coal deposit site were found to be inhabited by indigenous aerobic bacteria of the following genera: *Rhodococcus*, *Streptomyces*, *Arthrobacter*, *Pseudomonas*, and *Sphingobacterium*. This is a new finding expected to broaden our general knowledge about unique environmental habitats for specifically adapted microorganisms. From among the six isolates, four strains proved to be able to thrive in the presence of several aliphatic and aromatic hydrocarbons and to efficiently biodegrade these xenobiotics. This high biotransformation potential was revealed upon testing under fully aerobic conditions. The observed metabolic versatility of the isolated autochthons was confirmed by confronting the obtained results with the literature data regarding other bacterial biodegraders. Therefore, the biochemically robust lignite-colonizing strains are considered suitable candidates for bioaugmented reclamation of sites polluted with recalcitrant organic compounds. Further investigations should focus on the development of mixed microbial consortia to evaluate potential synergistic effects. Such consortia could increase the overall degradation rates of a wide spectrum of degradable compounds.

## Figures and Tables

**Table 1:** List of the strains isolated from lignite.

Strain Code	Identification	Identification Method	NCBI Accession Number (If Applicable)	The Closest Species in NCBI GenBank, Genome Accession Number (% of Identity) *
CUP1	*Streptomyces* sp.	16S rDNA	KT935296	*Streptomyces cinnabarinus* DSM 40467 CP114413 (99.15%)
CUP11	*Rhodococcus opacus*	16S rDNA	KT935299	*Rhodococcus opacus* C1 CP137577 (99.65%)
CUP12	*Arthrobacter* sp.	16S rDNA	KT935300	*Arthrobacter* sp. KBS0702 CP042172 (99.86%)
CUP15	*Pseudomonas fluorescens*	API 32GN, 16S rDNA	- **	*Pseudomonas fluorescens* NCIMB 11764 (98.80%)
CUP16	*Sphingobacterium* sp.	16S rDNA	- **	*Sphingobacterium* sp. VA-15b (98.52%)
CUP17	*Rhodococcus* sp.	16S rDNA	KT935301	*Rhodococcus erythropolis* KB1 CP050124 (100%)

* The data refer to the information collected in the NCBI GenBank database (November 2025). ** Accession numbers not available due to relatively low identification factors (identity < 99%). The 16S rDNA sequences are publicly available at https://www.ncbi.nlm.nih.gov/nuccore/“accession+number” (accessed on 27 November 2025).

**Table 2:** Qualitative results of the TTC-based respiratory test obtained after one-week observations of bacterial isolates incubated with xenobiotics applied at 1 g/dm^3^ initial concentration.

Strain	Benzoic Acid	Naphthalene	Phenanthrene	Pristane	Squalane	Hexadecane	Diesel Oil
*Streptomyces* sp. CUP1 *					+	+	
*Rhodococcus opacus* CUP11	+	+	+	+	+	+	+
*Arthrobacter* sp. CUP12 *			+			+	
*Pseudomonas fluorescens* CUP15		+				+	
*Sphingobacterium* sp. CUP16					+	+	
*Rhodococcus* sp. CUP17					+	+	+

Results marked “+” were found positive upon at least three out of four sample repetitions. * The strains were excluded from further bioremediation experiments due to poor growth.

**Table 3:** Xenobiotic concentration (g/dm^3^) changes during 14-day bioremediation experiment with selected strains.

Strain	Initial Xenobiotic Concentration (g/dm^3^)	Start	Day 3	Day 7	Day 14
**Diesel oil**					
Abiotic control	1.0	1.00 ± 0.50	1.05 ± 0.52	0.51 ± 0.25	0.52 ± 0.26
*Rhodococcus opacus* CUP11	1.0	1.03 ± 0.01	0.15 ± 0.01	0.04 ± 0.01	0.00 ± 0.01
	2.5	2.76 ± 0.35	1.40 ± 0.37	0.74 ± 0.13	0.56 ± 0.04
	5.0	6.14 ± 0.20	3.28 ± 0.95	2.14 ± 0.23	2.00 ± 0.58
	10.0	11.89 ± 0.25	9.63 ± 0.96	7.59 ± 0.14	6.36 ± 0.38
*Rhodococcus* sp. CUP17	1.0	0.99 ± 0.06	0.09 ± 0.01	0.05 ± 0.01	0.04 ± 0.01
	2.5	3.06 ± 0.35	0.37 ± 0.05	0.36 ± 0.02	0.12 ± 0.01
	5.0	5.94 ± 0.54	0.95 ± 0.23	0.35 ± 0.07	0.35 ± 0.01
	10.0	10.31 ± 1.05	2.68 ± 0.01	1.34 ± 0.01	1.00 ± 0.01
**Hexadecane**					
Abiotic control	1.0	0.73 ± 0.06	0.68 ± 0.03	0.85 ± 0.06	0.76 ± 0.16
*Rhodococcus opacus* CUP11	1.0	1.13 ± 0.07	0.23 ± 0.03	0.02 ± 0.02	0.01 ± 0.01
	2.5	2.71 ± 0.34	1.37 ± 0.36	0.74 ± 0.13	0.56 ± 0.04
	5.0	6.02 ± 0.20	3.21 ± 0.93	2.10 ± 0.23	2.00 ± 0.58
	10.0	11.62 ± 0.24	9.41 ± 0.94	7.42 ± 0.14	6.21 ± 0.37
*Sphingobacterium* sp. CUP16	1.0	0.86 ± 0.08	0.81 ± 0.12	0.71 ± 0.08	0.69 ± 0.10
*Rhodococcus* sp. CUP17	1.0	1.70 ± 0.17	0.15 ± 0.07	0.02 ± 0.01	0.01 ± 0.01
	2.5	3.36 ± 0.15	1.14 ± 0.08	0.10 ± 0.05	0.03 ± 0.02
	5.0	4.61 ± 0.77	3.33 ± 0.80	1.62 ± 0.19	1.86 ± 0.92
**Squalane**					
Abiotic control	1.0	1.00 ± 0.06	1.13 ± 0.19	1.13 ± 0.09	1.20 ± 0.11
*Sphingobacterium* sp. CUP16	1.0	1.67 ± 0.15	1.27 ± 0.12	1.26 ± 0.14	1.34 ± 0.12
*Rhodococcus* sp. CUP17	1.0	1.29 ± 0.27	1.41 ± 0.02	1.10 ± 0.01	1.13 ± 0.01
**Pristane**					
Abiotic control	1.0	1.13 ± 0.25	1.34 ± 0.03	1.30 ± 0.07	1.31 ± 0.05
*Rhodococcus opacus* CUP11	1.0	1.47 ± 0.04	1.27 ± 0.02	1.12 ± 0.03	0.89 ± 0.01
**Naphthalene**					
Abiotic control	1.0	0.82 ± 0.15	0.63 ± 0.04	0.54 ± 0.21	0.43 ± 0.12
*Rhodococcus opacus* CUP11	1.0	1.16 ± 0.05	0.84 ± 0.03	0.47 ± 0.10	0.28 ± 0.07
*Pseudomonas fluorescens* CUP15	1.0	1.28 ± 0.07	0.88 ± 0.13	0.72 ± 0.08	0.37 ± 0.05
**Phenanthrene**					
Abiotic control	1.0	1.03 ± 0.11	1.03 ± 0.07	1.08 ± 0.09	1.11 ± 0.10
*Rhodococcus opacus* CUP11	1.0	1.09 ± 0.06	1.15 ± 0.04	0.92 ± 0.03	0.99 ± 0.03

Variants depicted as “Abiotic control” represent uninoculated samples supplemented with a given xenobiotic at initial concentration of 1 g/dm^3^. Data are presented as mean values ± SD; n = 3.

**Table 4:** Stock solutions and amounts of xenobiotics used in bacterial screening test.

Xenobiotic	Stock Solution in Hexane (g/dm^3^)	Volume Transferred to the Well (cm^3^)	Volume Transferred to the Erlenmeyer Flasks (cm^3^)
Benzoic acid	6.67	0.030	-
Naphthalene	40	0.005	0.575
Phenanthrene	6	0.033	3.833
Pristane	20	0.010	- *
Squalane	40	0.005	0.575
Hexadecane	40	0.005	- *
Diesel oil	40	0.005	- *
Glucose	200 (in water)	0.001	0.115

* Xenobiotics applied in bioremediation tests directly at appropriate volumes.

## Data Availability

The raw data supporting the conclusions of this article will be made available by the authors on request. The 16S rDNA sequences of the strains reported in this article are publicly available in the NCBI GenBank database under accession numbers KT935296, KT935299, KT935300, KT935301.

## Abbreviations

The following abbreviations are used in this manuscript:

BH	Bushnell Haas medium
CFU	Colony forming unit
GC-MS	Gas chromatography coupled with mass spectrometry
LOD	Limit of detection
NCBI	National Center for Biotechnology Information
OD	Optical density
PAHs	Polycyclic aromatic hydrocarbons
rDNA	Ribosomal DNA
rpm	Revolutions per minute
SNB	Standard Nutrient Broth
TF	Tetrazolium formazan
TTC	2,3,5-triphenyltetrazolium chloride
